# The Potential Role of Microalgal Antioxidant Molecules on the Microbiota–Gut Axis in Inflammatory Bowel Diseases

**DOI:** 10.3390/biom15111535

**Published:** 2025-10-31

**Authors:** Rosa Paola Radice, Valeria Iannelli, Francesca Padula, Vincenzo De Fabrizio, Marios Drosos, Antonio Scopa, Giuseppe Martelli

**Affiliations:** 1 Department of Basic and Applied Sciences (DISBA), University of Basilicata, via Dell’ateneo Lucano 10, 85100 Potenza, Italy; francesca.padula@unibas.it (F.P.); giuseppe.martelli@unibas.it (G.M.); 2 AlgaeBioMed Srl, via Luigi Kossuth 7, 00149 Rome, Italy; 3 Department of Agricultural, Forest and Environmental Sciences (DAFE), University of Basilicata, via Dell’ateneo Lucano 10, 85100 Potenza, Italy; valeria.iannelli@unibas.it (V.I.); marios.drosos@unibas.it (M.D.); antonio.scopa@unibas.it (A.S.); 4 Department of Pharmacy (DIFARMA), University of Salerno, 84084 Fisciano, Italy; defabriziov@gmail.com

**Keywords:** microbiota, immune system, microalgae, nutrition

## Abstract

Comprising multiple microorganisms, the microbiota plays a crucial role in regulating the immune system and maintaining homeostasis. The influence of genetic and environmental factors causes the composition of the microbiota to change throughout life, which is called the plasticity of the microbiota. A eubiotic microbiota promotes the immune response, reducing the risk of inflammation and diseases such as IBD and cancer. The Mediterranean diet is of fundamental importance for a healthy microbiota. On the contrary, Western diets lead to microbiota dysbiosis and inflammation. Microalgae, and, in particular, their derivatives, show promise and relevance in the search for potential anti-inflammatory and antioxidant biomolecules. This review focuses on the correlation between microbiota, nutrition, immunity and microalgal derivatives, highlighting how these may be a potential innovative therapeutic strategy for the management of chronic inflammatory diseases.

## 1. Introduction

**Figure 1:**
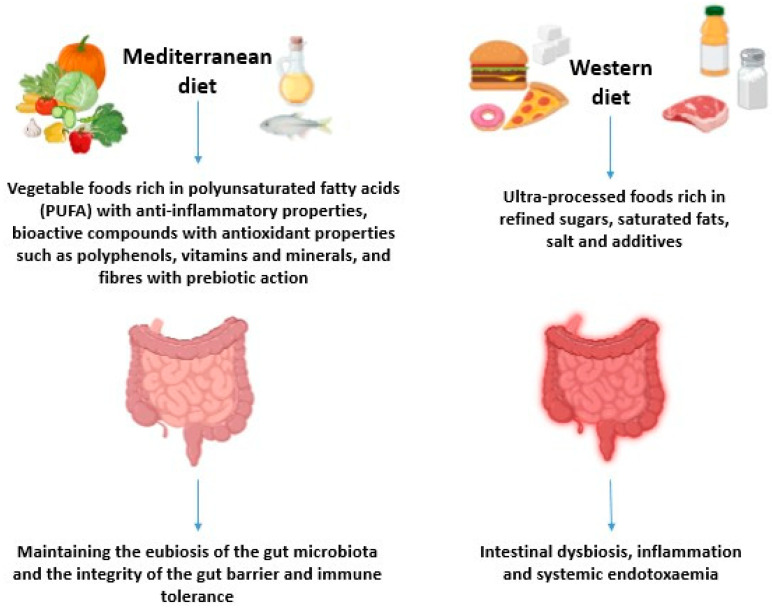
The Mediterranean diet involves the maintenance of the gut microbiota eubiosis and the integrity of the gut barrier and immune tolerance. West diets and ultra-processed foods result in the manifestation of permeable gut syndrome, gut dysbiosis, local inflammation and the presence of LPS in the bloodstream that will contribute to systemic endotoxemia and chronic inflammation [32].

The human body is composed of multiple microorganisms called the microbiota. The term microbiome, instead, defines the set of genes that the microbiota can express [1]. It is estimated that there are 10 to 100 trillion microbes in the gut, acquired at birth when the newborn is exposed to an environment rich in microorganisms. This exposure is important for the efficient maturation of the immune system [2]. Microbial colonization in the gut begins at birth, changes with age, and stabilizes during the lifetime. This highlights how the microbiota evolves throughout life and mutates. For this reason, the scientific community refers to microbiota plasticity [3]. Several factors can influence the microbial population and its stability, such as genetics, age, gender, nutrition, different diseases, the environment, and diet [4,5]. In particular, the Mediterranean diet is one of the external factors involved in maintaining microbiota homeostasis and preventing several diseases [6]. Conversely, the Western diet alters the microbiota and the immune system, contributing to the increase of various diseases [7] (Figure 1). Intestinal microorganisms are essential for maintaining health by producing fatty acids and participating in glucose and lipid metabolism. They are also involved in the differentiation of immune system cells [8]. The maintenance of homeostasis is of paramount importance, and its loss can lead to various diseases. Recent studies have shown a correlation between changes in the microbiota and diseases such as diabetes, colorectal cancer, and inflammatory bowel disease [9]. Given the enormous importance of the microbiome for human health, research groups are trying to identify the interactions between microbiota and gastrointestinal diseases, with a particular focus on their effects on the immune system [10]. Inflammatory bowel disease (IBD) is a pathological condition caused by a complex interaction of environmental, immunological, and genetic factors, which leads to chronic inflammation of the gastrointestinal tract. Among immunoregulatory molecules, the etiological agents responsible for oxidative stress, such as reactive oxygen species (ROS) and reactive nitrogen species (RNS), are overproduced in IBD. Among the various treatments currently used for IBD therapy, a new area of research is the use of antioxidant agents. Among these, astaxanthin (AXT), a powerful antioxidant carotenoid, is noteworthy for its ability to regulate oxidative stress and inflammation, produced by many plants and microalgae, especially *Haematococcus lacustris* (formerly known as *Haematococcus pluvialis*) [11]. Microalgae, in turn, represent a promising source of bioactive compounds with therapeutic value. The innovative use of microalgae derivatives offers a possible therapeutic option for IBD, based on protecting intestinal cells from oxidative stress and modulating inflammatory reactions.

## 2. Materials and Methods

To identify relevant studies and publications, a literature search was conducted in PubMed (MEDLINE), Google Scholar, and Scopus databases, using keywords such as “algae,” “microalgae,” “inflammatory bowel disease,” “IBD,” “Crohn’s disease,” “ulcerative colitis,” “intestinal inflammation,” and “astaxanthin.” To increase the amount of relevant information, artificial intelligence (https://scite.ai, accessed on 14 July 2025) was used to explore and delve deeper into specific topics, expand the relevant literature, and more critically consider the various involved paths. Extracted data and relevant results were selected based on publication relevance, methodology, and search results.

A total of 285 publications were selected, including systematic reviews, research articles, and meta-analyses.

## 3. Microbiota and Diet

Trillions of microbes inhabit the human body. The microbiota is defined as the set of microorganisms that are quantitatively and qualitatively present in a specific environment, such as the human gastrointestinal tract, which begins in the oesophagus and ends in the anus [9]. Over the past decade, the role of the gut microbiota has attracted significant research interest due to its complexity and involvement in human health and disease. Factors influencing the richness and diversity of the human microbiota include geographical location, age, genetic composition, hormonal changes, mode of delivery at birth (caesarean or vaginal), antibiotic use, lifestyle, and diet during childhood and adulthood [4]. The plasticity of the microbiota is greatest at birth when microbial colonization of the gut begins; during childhood there is a gradual increase in microbial diversity, and with advancing age, it becomes less malleable and stabilizes. Breastfeeding plays a significant role in microbiome formation, with the predominance of certain species such as *Bifidobacterium* and *Bacteroides*. Industrialization and unhealthy lifestyles in human populations are closely associated with higher rates of horizontal gene transfer (HGT). This mechanism consists of the introgression of exogenous gene fragments dynamically, between cells that are not direct progeny, conferring functional novelty to the genome into which it is inserted. Research studies have shown that HGT frequently occurs within individual gut microbiomes, and gut bacteria thereby acquire new functionalities. Modernity has led to increased hygiene, consumption of processed foods, reduced seasonality, and excessive use of antibiotics, continuously altering the gut microbiome [12]. The maternal microbiome shapes the infant’s gut microbiome through vertical transmission at birth and horizontal gene transfer events. In particular, an interspecies transfer of mobile genetic elements from mother to infant has been discovered. The main genes identified are associated with diet and are involved in encoding functions related to carbohydrate utilization, amino acid metabolism, and iron acquisition and storage. Mother–infant HGT may have consequences not only on the gut microbiome but also on the development of the immune system [13]. The human host and microbiota have co-evolved, forming a symbiotic relationship. The host provides a place for growth, suitable conditions, and nourishment for gut bacteria, while the microbiota supports bodily functions, induces resistance to infection, and facilitates the absorption of ingested food. The microorganisms that constitute the gut microbiota belong to the genera *Bacteroidota*, *Bacillota*, *Pseudomonadota*, *Actinomycetota*, *Fusobacteria*, and *Verrucomicrobia*. Recent studies confirm that in most individuals, the microbiota can be classified into three main groups: *Bacteroides*, *Prevotella*, and *Ruminococcus* [14]. The function of the gut microbiota is to protect against pathogens through colonization of mucosal surfaces, production of antimicrobial substances, enhancement of the immune system, regulation of digestion and metabolism, proliferation and differentiation of epithelial cells, communication, and brain–gut functions [15]. The plasticity of the microbiota can be summarized in six stages.

- Prenatal phase: the hypothesis of colonization in utero has been questioned, although analysis is difficult due to limited quantities of microbes and the analytical limitations of 16SrRNA sequencing.
- Perinatal phase: the microbial pattern is established depending on the mode of delivery. With vaginal delivery, maternal vaginal microbes colonize the infant’s gut, with an enrichment of *Bifidobacterium* spp. and a reduction of *Enterococcus* and *Klebsiella* spp. compared to cesarean section delivery, where the infant’s gut is mainly colonized by skin microbes. These differences may disappear within a year of life.
- Postnatal phase: the composition of the gut microbiota changes depending on the consumption of breast milk or artificial milk and markedly with weaning [16].
- Childhood and adolescence: the pediatric gut microbiota has similar characteristics to that of adults, but a child’s gut hosts larger amounts of *Firmicutes* and *Actinobacteria* and smaller amounts of *Bacteroidetes* than the adult microbiota.
- Adulthood: an equilibrium between the host and the microorganisms in the gut is reached, it can be changed through diets and the use of drugs such as antibiotics. After treatment with antibiotics, restoration of the original microbial composition can take place with the use of probiotics. Autologous fecal microbiota transplantation (FMT), on the other hand, restores the gut microbiota within a few days after antibiotic administration.
- Older age: increased susceptibility to infections may emerge due to an altered immune system leading to chronic low-level inflammation and dysbiosis in the gut microbiota causing pathogen growth and disease outbreaks. Studies conducted on healthy older individuals suggest the maintenance of a good microbial composition over time ensuring longevity and healthy aging [3].

The gut microbiota plays a decisive role in host homeostasis and numerous gastrointestinal diseases result from imbalances in the composition of the microbiota. Qualitative and quantitative alterations of the gut microbiota are observed with irritable bowel syndrome (IBS). The main immune-mediated chronic inflammatory diseases (IBD) affecting the digestive system are ulcerative colitis (UC) and Crohn’s disease (CD). *Desulfovibrio* species are abundant and *Escherichia coli* and low levels of *F. prausnitzii*, *Bacteroidetes*, and *Firmicutes*. The onset and development of IBD depend on several factors, including genetic susceptibility with a total of 199 specific genes identified for IBD, immune factors, and the gut microbiota. A high risk of developing IBD has been observed in the offspring of affected parents. In IBD, the reduction in the content and diversity of the gut microbiota weakens the microbial barrier, with an increased risk for opportunistic pathogens to invade the intestinal mucosa, leading to an immune disruption between Th17 and Treg cells, aggravating the inflammatory phenomenon. In affected individuals compared to healthy individuals, the content of metabolites such as SCFA (short-chain fatty acids), and primary and secondary bile acids (BAs) in the colon is drastically reduced and the metabolism of tryptophan is altered [17]. Increased intestinal permeability plays a role in the pathogenesis of IBD. Probiotic supplementation and fecal microbiota transplantation (FMT) can be used to treat IBD [18]. Chronic intestinal inflammation has also been associated with the development of colorectal cancer (CRC) and the increased involvement of *Bacteroides fragilis* and *Streptococcus bovis*, activating immune cells to release pro-mitogenic and pro-angiogenic cytokines such as Interleukin-17 (IL-17) and the synthesis by the gut microbiota of metabolites involved in carcinogenesis such as secondary transformations of bile salts, desulphurization of bile acids, synthesis of aromatic amines by azoreductase and nitroreductase, formation of reactive oxygen species [19]. In recent years, the role of the gut microbiota as a risk factor for the development of metabolic syndrome has been discovered. Hyperglycaemia, hypertension, and dyslipidemia are related to dysbiosis of the gut microbiota [20]. There is a close relationship between circadian rhythms, gut microbiota, and metabolic dysfunction associated with the West lifestyle in promoting carcinogenesis and cancer development, particularly colorectal cancer [21]. One of the methods used to characterize the gut microbiome is stool sample analysis. DNA extraction involves purification of the sample with reagents, lysis of the bacterial cells with lysis buffer, amplification, and sequencing of the 16S amplicon (hypervariable regions of 16S ribosomal RNA). The sequencing reads are grouped into operational taxonomic units (OTU clustering algorithm) and based on sequence homology to the 16S rRNA gene databases it is possible to identify bacterial genera and species [22]. Microbial diversity plays an important role in human metabolism and their potential use in the treatment of infectious and chronic diseases and therapeutic applications [8].

Diet is the main modulator of the functions and composition of the gut microbiota. A balanced diet can ensure the formation of a good microbial flora, where all bacterial species coexist in a mutually balanced system [23]. During birth there is colonisation of the gut, which changes throughout life in microbial diversity and abundance. Many studies have shown that breastfeeding is the strongest predictor of the composition of the gut microbiota in the first months of life, promoting healthy development of the immune system and preventing pathological imprinting. A marked transformation in the gut microbial community occurs after weaning with the introduction of solid foods. The use of antibiotics has a significant impact on the evolution of the infant gut microbiota by increasing Proteobacteria and reducing Actinobacteria populations, decreasing overall diversity, and selecting drug-resistant bacteria. Epidemiological studies have shown that antibiotic consumption in early childhood leads to an increased risk of allergic diseases [24]. In early childhood, commensal intestinal microbes such as *Bifidobacterium infantis*, *Bifidobacterium bifidum*, and *Lactobacillus rhamnosus* can enhance the development of the intestinal barrier by strengthening tight junctions in intestinal epithelial cells. *Bacteroides thetaiotaomicron* and *Lactobacillus reuteri* attenuate host inflammatory responses by inhibiting activation of nuclear factor-κB (NF-κB) [25]. The process of urbanization has significantly shaped the gut microbiota, thus affecting the functionality of the gut microbiome. Each person’s microbiome is unique, develops rapidly during early childhood, and is relatively stable but susceptible to changes in adulthood depending on the type of diet followed. The Mediterranean diet (MD) is a dietary pattern that exerts a beneficial role on the gut microbiota in particular on its diversity and metabolic activities, promoting a state of eubiosis in terms of balance and well-being of the microbiota with a predominance of beneficial microorganisms. It is characterized by a high content of plant-based foods and habitual consumption of cereals, legumes, olive oil and nuts. These foods provide polyunsaturated fatty acids with anti-inflammatory properties, a variety of bioactive compounds with antioxidant properties such as polyphenols, vitamins, and minerals [6]. Fibre has a prebiotic action for the microbial growth of bacteria living in the human gut and is an excellent substrate for the metabolism of bacteria to produce SCFAs (short-chain fatty acids) mainly butyrate, propionate, and acetate. These metabolites play an important role in the regulation of immunity [26]. In contrast, a West diet high in saturated fats, refined carbohydrates, and animal protein, low in plant/fruit foods, and low in omega-3 PUFAs, can have a direct effect on the immune system causing intestinal barrier disruption, metabolic disorders and dysbiosis [27]. Dysbiosis is a structural and functional change in the gut microbiota, which can trigger severe inflammation through an increased number of pro-inflammatory microorganisms. Moreover, dysbiosis is directly related to the onset of colorectal cancer (CRC) [26]. A dysbiosis in the gut microbiota can lead to a reduced ability to induce a local and systemic immune response, resulting in local inflammatory diseases and diseases at distal sites [28]. The gut microbiota can produce metabolites through the fermentation of ingested food. The metabolites act as substrates and regulate the activities of epigenetic modification enzymes (DNA methylation, histone modifications and the expression of non-coding RNAs), influencing the expression of host genes and triggering immune inflammation in intestinal epithelial cells (IECs), resulting in metabolic disturbances through altered intestinal permeability [29]. A diet rich in whole grains, fruit and vegetables has beneficial effects on the bacterial composition, and reduces opportunistic bacteria, resulting in a reduction of lipopolysaccharide (LPS), trimethylamine-N-oxide (TMAO) and inflammatory cytokines. Conversely, increased SCFA production reduces inflammation and the risk of obesity and type II diabetes [30]. Diet can have a direct effect on host physiology and health and an indirect effect by altering the composition of the gut microbiota and/or its production of metabolites that in turn impact host physiology causing disease. Recent studies in animal models show a cause–effect relationship between diet and host health via the gut microbiota. Examples include strengthening barrier function through SCFA production; protection of the mucus layer through fiber acting as a nutritional substrate for the microbiota; SCFA increasing regulatory T-cells supporting immune tolerance and GALT homeostasis [31]. In addition, tryptophan metabolites such as indoles are derived from commensal fermentation of dietary tryptophan and function as ligands for the aryl hydrocarbon receptor (AhR), a receptor important in maintaining intestinal homeostasis. Loss of these metabolites is associated with the onset of inflammatory bowel disease. Nutritional intervention is a potential therapeutic approach, targeting both the gut microbiota and the immune system. Several studies discuss the relevance of immuno-nutrition, the ability to modify nutrient intake to modulate immune responses [32].

Based on numerous research studies, it is speculated that a high-fat diet may promote gastrointestinal tumors by altering the composition of gut microbes [33]. For example, high-fat diets can lead to increased production of bile acids converted by intestinal bacteria to secondary bile acids (deoxycholic acid and lithocholic acid). Due to their genotoxic and pro-inflammatory effects, they have been implicated in the promotion of both colon and liver cancer. Conversion may also result in an immunomodulatory role in promoting liver cancer progression by inhibiting the recruitment of anti-tumor natural killer T cells in the liver [34]. In the last few years, numerous investigations revealed deep modifications of intestinal microbiota composition, firmly linked with lifestyle and altered metabolic status. More precisely, Lynch and Pedersen point out how the reduction of bacterial diversity and the depletion of beneficial species such as *Faecalibacterium prausnitzii* and *Akkermansia muciniphila* are accompanied by an expansion of bacteria typical of inflammatory and obesogenic disorders [35]. These alterations have a deep impact on systemic metabolism, like that of oxylipins, oxidative metabolites of polyunsaturated fatty acids (PUFAs) playing an important role in the regulation of inflammation and lipid homeostasis. Dysbiosis induced by high-fat diet alters the plasma oxylipin profile with an increase of pro-inflammatory arachidonic acid derivatives (e.g., 5-HETE, 12-HETE) and a reduction of anti-inflammatory omega-3 fatty acid-derived products (e.g., 17-HDHA, 14-HDHA) [36]. These changes are linked with the decrease of SCFA-producing bacteria, cementing the interaction between microbiota, lipid metabolism, and oxidative regulation. This microbiota–oxylipin axis explains the formation of a chronic, low-grade inflammatory microenvironment that is typical of obesity as well as other metabolic syndromes [37,38]. Intestinal dysbiosis alters not only microbial metabolite synthesis but also oxylipin biosynthesis and functional balance, regulating key inflammatory and metabolic processes. This supports the idea of a bidirectional dialogue between microbiota and oxidative lipid metabolism that is a potential therapeutic target in obesity and chronic inflammatory disease states [39,40]. The most important factor in preventing and fighting cancer is a well-functioning immune system. This requires a good nutritional status allowing the gut microbiome to play all its roles in metabolic control, immune control and communication with other organs [40,41].

## 4. IBD

The coexistence of the microbiota and the host is essential for maintaining vital host functions, and disruption of this complex coexistence has been associated with several diseases, such as IBD. IBD, which includes Crohn’s disease and ulcerative colitis, differs in symptoms, location, and histopathological features. It is characterized by chronic inflammation of the gastrointestinal tract and a clinical course alternating between phases of remission and relapse, causing significant morbidity and reduced quality of life in patients [41,42,43,44,45]. The pathogenesis of IBD is complex and still not fully understood, although it is characterized by a combination of genetic susceptibility, dysbiosis, immune system dysregulation, and environmental factors [46,47]. Recent research has highlighted the interaction between gut microbiota, immune system and genes in the pathogenesis of IBD [4]. The intestinal microbiota, in particular, has been identified as a determining factor, capable of influencing susceptibility to the disease and response to therapy [48,49]. Dysbiosis, caused by reduced microbial diversity, increased epithelial permeability, loss of anti-inflammatory bacteria and increase of pro-inflammatory ones, is a key element [50,51]. Precisely for this reason, technological advances in metagenomic sequencing have contributed to a greater understanding of microbial dysbiosis in IBD, paving the way for therapies targeting the microbiota [52,53,54]. Chronic inflammation associated with oxidative stress causes a disorganized immune response and an imbalance of the gut microbiota [55]. The role of cytokines, important in the pathogenesis and inflammatory mediation, has been extensively studied and many targeted therapies have been developed with positive outcomes in several clinical trials [56,57,58]. The imbalance between pro- and anti-inflammatory mediators is also mainly influenced by a redox imbalance, where the imbalance between ROS/RNS production and endogenous antioxidant defenses is an important pathogenic factor for these diseases [59,60,61,62]. ROS and RNS, although involved in physiological antimicrobial defense, cause oxidative damage to cellular structures, aggravating the inflammatory process and are mainly generated by activated immune cells (macrophages, neutrophils) and intestinal epithelial cells in response to pro-inflammatory stimuli such as TNF-α, IL-1β and IL-6 [63,64]. Macrophages and dendritic cells of the lamina propria, when they recognize microbial antigens via receptors such as TLR and NOD2, release proinflammatory cytokines (TNF-α, IL-1β, IL-6, IL-12/23), which recruit neutrophils and activate helper T cell responses [65,66,67,68]. In parallel, the function of Foxp3+ regulatory T cells (Treg) is compromised, losing the ability to maintain immune tolerance [69,70]. In CD, a Th1/Th17 response predominates, with increased IFN-γ and IL-17, while in UC a Th2 response is observed, with increased release of IL-4 and IL-13 [71,72,73,74,75]. The inflammatory cascade in IBD is always associated with excessive production of ROS and RNS, particularly superoxide anion (O_2_^−^), hydroxyl radical (OH) and nitric oxide (NO) [76,77]. Their overproduction compromises the integrity of the intestinal barrier, thus increasing intestinal permeability, which favors bacterial translocation, which in turn further activates immune cells and strengthens inflammation [78,79,80,81,82,83,84,85]. The interaction between O_2_^−^ and NO produces peroxynitrite (ONOO^−^), a highly damaging reactive nitrogen species, which causes protein nitrification and further tissue damage [86,87]. In particular, peroxynitrite activates proinflammatory signals such as NF-κB and MAPK, increasing the secretion of cytokines [87,88]. These redox environments further regulate the immune response through NLRP3 inflammasome activation, Th1/Th17 polarization, Treg dysfunction, and mitochondrial damage [25,59,60]. Mutations in the NOD2 and ATG16L1 genes interfere with autophagy and antimicrobial defense processes, leading to increased production of proinflammatory cytokines and impaired integrity of TJs [61,62,63]. This promotes the penetration of antigens and the consequent activation of the immune response [47,89]. Dysbiosis and genetic predisposition close the vicious circle, fueling inflammation and tissue damage [90,91,92]. Foxp3+ Treg cells, particularly the RORγt+Foxp3+ subpopulation, are essential for avoiding excessive inflammation in the gut [93]. Their significant depletion has been demonstrated in the colonic lamina propria in murine models of IBD, suggesting that their loss contributes to chronic inflammation [94,95,96]. Dysbiosis, in fact, compromises immune homeostasis, as occurs, for example, in invasive-adhesive Escherichia coli (AIEC), a pathobiont often associated with IBD, which exploits the host’s genetic predisposition (e.g., polymorphisms in NOD2 and ATG16L1) to escape immune clearance and increase inflammation [97,98,99,100,101]. The intestinal epithelial barrier, protected by tight junction proteins such as claudins, is fundamental and its dysfunction, which results in increased permeability, allows luminal antigens to invade the lamina propria, activating the immune response [102,103,104,105]. Concomitantly, there is a deficiency in the production and function of regulatory T cells (Treg), particularly of the anti-inflammatory cytokines IL-10 and TGF-β, normally involved in maintaining immune homeostasis [106,107]. This situation facilitates the migration of neutrophils, macrophages and dendritic cells into the lamina propria, which release inflammatory mediators, ROS and RNS [107,108]. Dysbiosis and genetic predisposition close the vicious circle, fueling inflammation and tissue damage [90,91,92]. Foxp3+ Treg cells, particularly the RORγt+Foxp3+ subpopulation, are essential for avoiding excessive inflammation in the gut [93]. Their depletion has been demonstrated in the colonic lamina propria in murine models of IBD, suggesting that their loss contributes to chronic inflammation [94,95,96]. The intestinal epithelial barrier, protected by tight junction proteins such as claudins, is also fundamental [102]. Its dysfunction, in the form of increased permeability, allows luminal antigens to invade the lamina propria, activating the immune response [103,104,105]. Dysbiosis exacerbates the damage, as demonstrated by the reduction of microbial diversity and the proliferation of proinflammatory taxa [64,80,84]. The primary target of IBD therapy is TNF-α, and cyclical treatments with anti-TNF-α agents have been shown to reduce inflammation and induce remission [109,110]. Recently, research has also focused on blocking other cytokine pathways, such as IL-23 and IL-12, in the process of T-helper cell activation and differentiation, for example, with ustekinumab, as an alternative treatment for patients refractory to anti-TNF-α [111,112,113,114,115]. Traditional therapies, such as aminosalicylates, corticosteroids and immunosuppressants, have been the main therapeutic approaches, but they have significant side effects and unpredictable efficacy [116,117,118]. Despite their high cost, biological therapies have revolutionized the management of IBD, improving clinical outcomes in most patients while highlighting treatment refractoriness in some patients, underscoring the need for alternative and innovative therapeutic approaches. [119,120]. Recent studies suggest that redox balance intervention through antioxidant treatments can reduce inflammation in IBD [121]. To this end, carotenoids have attracted the attention of the scientific community as adjuvants in the treatment of IBD [122,123]. Carotenoids are a highly diverse class of pigments that play an important role in the biological processes of plants, algae and some bacteria, contributing to the bright colours of fruits and vegetables and participating in photosynthesis [124,125]. Carotenoids have long been recognized for their single oxygen neutralization and free radical scavenging activity, thus protecting cells from oxidative stress [126,127,128]. Their activity is particularly relevant in chronic diseases such as cancer, diabetes, obesity, cardiovascular and neurodegenerative diseases, where oxidative stress and inflammation are key factors [76,129,130]. In recent years, there has been a growing interest in xanthophylls and microalgae, a subclass of carotenoids that includes astaxanthin (AXT), lutein and zeaxanthin [131]. AXT, in particular, has attracted great interest due to its antioxidant and anti-inflammatory properties, making it a suitable candidate for the treatment of inflammatory diseases such as rheumatoid arthritis [132,133]. In addition to their antioxidant and anti-inflammatory actions, carotenoids also contribute to intestinal health [134,135]. The interaction between carotenoids and gut microbiota is a new area of research, with important therapeutic implications for IBD [134,135,136]. Dietary interventions, particularly those rich in carotenoids, have shown potential benefits in modulating intestinal inflammation and improving clinical response in patients with IBD [137,138].

## 5. Microbiota and Immune System

**Figure 2:**
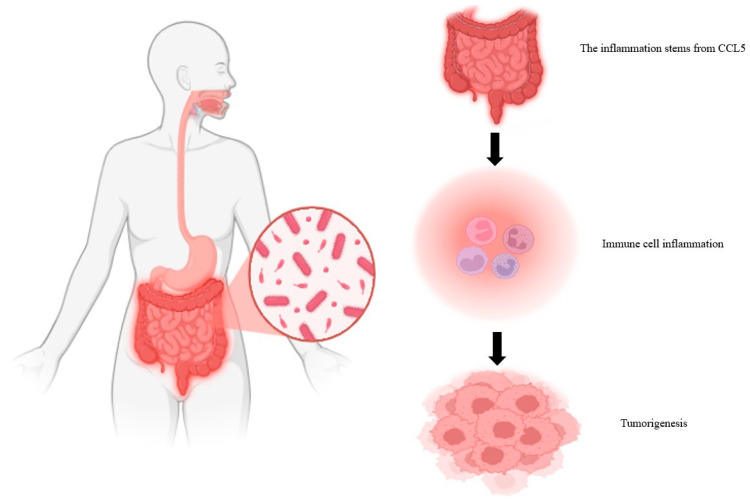
Tumor development from the inflammatory state [153].

The microbiota consists of a series of microorganisms (viruses, bacteria, fungi) that cooperate with the host by regulating various functions including the immune response to pathogens. To ensure homeostasis, gut bacteria use molecules produced during their replication to control microbial population density and gene expression. Alterations in the microbiota cause inflammation, infection and disease onset [28]. The organ that hosts the most immune cells is the gut. This has dendritic cells, macrophages, T-cells and B-cells capable of inducing an immune response against pathogens without altering the microorganisms present [139]. Bacteria in the gut interact with the immune system by conditioning the maturation of immune cells, regulating the immune response and supporting its development in childhood [140]. Microbial occupation begins simultaneously with the maturation of the immune system during childhood. The microbiota–immune system correlation is crucial for immune tolerance and the prevention of various diseases [49]. The bacteria in the gut produce vitamins, fatty acids and various compounds essential for maintaining health. In addition to defending against the attack of pathogens, intestinal bacteria can keep the intestinal wall intact [141]. Furthermore, essential compounds such as vitamins are key in the innate and adaptive immune response. The loss of just one essential nutrient could alter immunity [142]. In detail, the production of fatty acids induces an enhancement of the anti-inflammatory response with the production of antibodies by B cells and an enhancement of the action of T cells [143]. Alterations in the microbiota are causative of alterations in the immune response resulting in disease. Underlying this is the restoration of homeostasis [144]. Also at the genetic level, through transcriptomics studies on adaptive and innate cells and through epigenetic regulation of cytokines, it has been possible to study the microbiota–immune system interaction. Studies in mice show that microbiota–immune system imbalance underlies multifactorial diseases, such as cancer, infectious diseases, metabolic diseases, diabetes, and inflammatory and neurodegenerative diseases [145]. Among the latter, relevant are Parkinson’s disease and Tourette’s syndrome [146]. The variety of diseases results from a variable microbial composition. For example, there are different microbial populations along the gastrointestinal tract. Each of these can be considered on its own and limited by nutrients and organs. Thus, alteration of one population may be causative of a given disease [147]. In a healthy subject, Toll Like Receptor (TLR) levels are low. These increase in the case of an imbalance between the microbiota and the immune system resulting in the activation of monocytes, macrophages, and natural killer cells involved in innate immunity [148]. Alteration of homeostasis resulting from disruption of the microbiota–immune system balance is causative of intestinal diseases. Underlying the imbalance is an inflammatory context. The continued presence of an inflammatory state can be causative of neoplasms [149]. The stability of the intestinal barrier is due to fermentation products obtained from the microbiota. In general, TLRs recognize bacterial-specific components that are presented to CD4 T cells and B cells [150]. The microbiota can induce the activation and differentiation of B cells resulting in the production of antibodies [151]. These can bind the antigen, blocking pathogenic action and thus infection. Alterations in the process can lead to an increase in pathogenic Gram-negative bacteria resulting in an alteration of the intestinal barrier and increased infection [150]. Among these, inflammatory bowel diseases are very common. In the altered microbiota–immune system correlation, both T-helper cells (pro-inflammatory) and regulatory T-cells (anti-inflammatory) are activated. Active T-helper cells produce cytokines and molecules causing inflammatory states with a proliferation of pro-inflammatory bacteria called pathobacteria [152]. Pathobacteria, through specific virulence mechanisms, can evade the immune system by generating toxins. The transformation of bacteria in the microbiota into pathobacteria induces a loss of beneficial responses in the body resulting in the risk of developing critical illnesses that can cause mortality [153]. Innate response cells regulate the homeostasis–inflammation transition and are involved in the development of cancer. In detail, in a tumor context, innate cells undergo functional changes and lose their ability to induce and aid the immune response [154]. The disruption of the balance of the intestinal microbiota, increasing pathogenic bacteria, is called intestinal dysbiosis. The resulting inflammatory state can be causative for tumor development. Inflammation arises from chemokine ligand 5 (CCL5), which attracts lymphocytes to the gut. Local inflammation induces the activation of interleukin-6 (IL-6) and the proliferation of intestinal epithelial cells resulting in tumor formation (Figure 2) [155].

Several studies have shown that certain gut bacteria can be used as biomarkers for digestive cancers such as pancreatic cancer and hepatocellular carcinoma. Treatments for gastrointestinal diseases include microbiota transplantation, the use of prebiotics and probiotics, and the possibility of using engineered bacteria [156]. Moreover, since the microbiota is able to induce the activation of anti-inflammatory and pro-inflammatory cells, its modulation can be effective in cancer immunotherapy [157].

## 6. Microbiota and Cancer

In 2020, there were 19.3 million cancer cases and 10 million deaths. Infection with *Helicobacter pylori*, a Gram-negative bacterium, is considered the leading cause of gastric cancer [158]. Tumor initiation and progression caused by infection could be induced by mutated gene expression and activation of signaling pathways [159]. It was observed that some tumor cases were attributable to other infectious contexts of papilloma virus and hepatitis B and C virus [160]. The cancer–microbiota interaction is complex and requires further study, but in about 20% of cancers, microorganisms are thought to be involved and take part in the tumor environment. Carcinogenesis is triggered by a disruption of the homeostasis of the colon epithelium due to both tumor cell proliferation and host cell death [161]. Tumor cells originate from acquired genetic modifications, undergoing uncontrolled proliferation and escaping immune control. Certain microorganisms can thus promote tumourigenesis by altering the immune response and creating a favorable environment for tumor development [162]. The gut microbiota and its bioactive metabolites do not directly cause cancer but may promote and modulate tumor progression and therapeutic efficacy [163]. Colorectal cancer (CRC) is considered one of the most devastating and widespread malignancies worldwide. CRC is the third most commonly diagnosed cancer, and a genetic predisposition underlies its development. In the cancer patient, the gut microbiota evolves with the pathogenesis of the disease, the etiology of the tumor being due to an infectious insult or high consumption of alcohol, smoking, obesity and diabetes [164]. The main tumor-promoting mechanisms are pathogenic bacterial virulence factors and/or toxins, bacterial metabolic products and dysregulation of immune responses [165]. Microbiota-related carcinogenesis is due to dysbiosis-related inflammation and carcinogen formation. Cancer-associated intestinal dysbiosis in individuals genetically predisposed to IBD can induce an aberrant immune response and the proliferation of pro-inflammatory bacteria, which increase inflammation leading to altered intestinal permeability [152]. A diet rich in saturated fats and simple sugars can induce microbial dysbiosis in the gut and increase the risk of CRC with the production of carcinogenic metabolites such as NOC (N-nitroso compounds), TMAO and secondary bile acids [166]. The most common bacterial species in CRC patients detected in biopsies is *F. nucleatum*, which besides being involved in inflammation interacts with E-cadherin expressed on both epithelial cells and tumor cells through its virulence factor FadA. This bacterium upregulates oncogenes and the subsequent phosphorylation of β-catenin results in the activation of NF-KB by upregulating the synthesis of inflammatory cytokines and cell proliferation [167]. Metabolites may inhibit or promote tumor development by inducing pro-inflammatory and immunosuppressive effects. Short-chain fatty acids (SCFAs) and bile acids such as ursodeoxycholic acid (UDCA) are the main metabolites of the gut microbiota with a protective role. They inhibit inflammation, promote apoptosis of cancer cells and defend colon epithelial barriers from cancerous invasion [168]. For example, butyrate has antitumor activity. Recent studies have found that the effects of butyrate vary depending on its concentration, high concentrations of butyrate inhibit tumourigenesis, while low concentrations promote tumor evolution; however, further studies are needed [169]. Some bile acids and TMAO, on the other hand, promote cancer cell proliferation, metastatic spread, DNA damage and genomic instability. Moreover, the presence of bacteria in the gastrointestinal tract induces the breakdown of heme leading to the production of hydrogen sulfide and nitric oxide, these compounds also cause DNA damage and promote carcinogenesis [170]. It has been reported how epigenetic alterations in the colorectal epithelium such as DNA methylation, histone modifications and non-coding RNAs are due to the gut microbiota, and its metabolites act as a potential driving force for the initiation and promotion of tumourigenesis in CRC [171]. Host-gut microbiota communication is required to maintain gut homeostasis through a regulatory network in which microRNAs (miRNAs) have emerged as major mediators. Diet-induced microbial metabolites can influence miRNA expression in host cells and promote or inhibit tumors in CRC. Host-derived miRNAs such as miR-515-5p and miR-1226-5p may also affect the microbiota and promote bacterial growth of *F.nucleatum* and *E. coli*, suggesting a possible influence on tumor growth by modifying bacterial activities and the gut microenvironment [172]. Patients with CRC and healthy individuals have different gut microbiota in their feces, which is why the gut microbiota can serve as a microbial marker to assess CRC risk and can be used to screen for precancerous lesions and CRC [173]. Also, the gut microbiota influences antitumor immunity through the production of inosine and hypoxanthine involving the proliferation of T-helper 1 cells by inducing the death of transformed cells, inhibition of inflammatory cytokines and blockade of checkpoint sites, which could be of interest in therapeutic applications [174]. Modulation of the gut microbiota represents an innovative and effective strategy in cancer treatments [175]. Precision and customized cancer treatments are possible by combining bacteria with nanotechnology, optimizing drug targeting and reducing toxic and side effects [176]. Numerous studies have shown improved health in cancer patients through the use of probiotics, which can inhibit tumor growth and modulate the individual’s inflammatory state [177].

## 7. Microalgae

In order to regulate oxidative stress and the immune response, interest in microalgae is growing significantly as potential sustainable alternatives. Microalgae are photoautotrophic plant organisms due to the presence of photosynthetic pigments, such as chlorophyll and carotenoids. Microalgae exhibit a high ability to colonize various environments and to respond very rapidly to environmental changes, making them organisms with remarkable adaptability thanks to their high metabolic plasticity [178]. Moreover, microalgae are extremely efficient organisms due to their excellent biochemical composition in:

- Proteins, which serve both biological and structural functions. Microalgae tend to accumulate proteins in amounts ranging from 10% to 70% of the dry biomass weight.
- Lipids and microalgal biomass contain both structural (polar) and storage (neutral) lipids. Microalgae are rich in long-chain fatty acids with up to 20 or 22 carbon atoms, and in polyunsaturated fatty acids (PUFAs) such as docosahexaenoic acid (DHA), eicosapentaenoic acid (EPA), and gamma-linolenic acid (GLA). The most relevant are omega-3 and omega-6 fatty acids. Ossilipins can be obtained from the oxidation of PUFAs. These are lipophilic signalling molecules mainly involved in inflammatory processes and stress responses [179]. The activity of new families of bioactive mediators derived from omega-3 polyunsaturated fatty acids, such as eicosapentaenoic acid and docosahexaenoic acid, is significant. These mediators, called resolvins, docosatriens and protectins, have protective and anti-inflammatory properties [180]. Their role is promising in the resolution of inflammation and, in general, in the development of new therapeutic approaches [181].
- Carbohydrates, the composition is species-specific in microalgae and includes both monomeric sugars (monosaccharides) and polymeric forms (di-, oligo-, and polysaccharides). The most abundant sugars are glucose, rhamnose, xylose, and mannose. They serve various functions such as storing metabolic energy and are also major structural components of the cell wall, along with glycoproteins. It is estimated that sugar content can account for up to 60% of the dry biomass weight.
- Photosynthetic pigments with potential applications in cosmetics, nutraceuticals, and pharmaceuticals. They exhibit remarkable properties, including antioxidant, anti-inflammatory, neuroprotective, hepatoprotective, anti-mutagenic, and anti-allergic effects. The three main classes of pigments are: phycobiliproteins (PBPs), carotenoids (carotenes and xanthophylls) and chlorophylls, the green pigments typical of plants [182].
- Vitamins, the accumulation and synthesis of vitamins in photosynthetic organisms are highly variable and closely linked to physiological responses to environmental changes. Microalgae are producers of many essential vitamins such as vitamin A, B-group vitamins, vitamin C, vitamin D, vitamin K, and vitamin E [183].

*Haematococcus lacustris* is a unicellular green microalga belonging to the *Chloropyta*. This is a great source of fatty acids and protein, and it is the largest producer of astaxanthin (AXT). AXT is a xanthophyll belonging to the red carotenoids [184]. Structurally, it has β-ionic rings containing oxygenated ketone and hydroxyl groups linked together by a polyene chain. For this reason, AXT has antioxidant properties and reduces the production of free radicals. AXT is widely used as a protective agent in many diseases caused by oxidative stress. In addition to its antioxidant properties, it is also an anti-inflammatory and anti-tumour agent [184]. Thanks to its properties, AXT is very interesting in the treatment of chronic inflammatory diseases such as IBD.

## 8. Potential Therapies and Biomolecules from Microalgae

Several classes of drugs, from glucocorticoids to biologics, are used for the treatment of IBD. Biological treatments have revolutionized disease management, targeting selective inflammatory molecules and improving the quality of life of many patients [185,186]. For a long time, IBD therapy has focused on controlling pain and associated symptoms, using various drugs such as aminosalicylates and corticosteroids, although the latter, while beneficial for many patients, have significant side effects [187,188,189]. However, treatment resistance and relapses remain common problems, highlighting the need for new or adjuvant therapeutic options [190]. Probiotics, as modulators of the intestinal microbiota, have been considered for their potential to restore intestinal homeostasis and reduce inflammation [191,192]. The production of probiotics with specific strains, such as *Limosilactobacillus fermentum*, represents an area of research to improve their therapeutic potential [193]. The synergistic use of probiotics with other compounds, such as hydroxyectoine, can amplify the beneficial effects on the intestinal environment. Hydroxyectoine, in fact, forms a protective and hydrating envelope around probiotic cells, stabilizing their proteins and membranes as they pass through the intestine, preventing damage caused by dehydration, acids, and bile salts, increasing the survival of probiotics and amplifying their beneficial effects on the intestinal environment [194]. Recent studies have identified new therapeutic molecules useful in the treatment of IBD such as oroxyloside, a chemical compound that improves DSS-induced colitis through the inhibition of endoplasmic reticulum stress by activating PPARγ and polyphenolic agents such as resveratrol and curcumin which have shown the ability to act on cellular signaling pathways and suppress intestinal inflammation [119,120,195,196,197]. These results indicate that the search for new molecules, natural or otherwise, can expand therapeutic options to restore intestinal homeostasis and improve the quality of life in IBD patients. For this reason, natural bioactive compounds are used as supplements or as therapeutic alternatives to alleviate IBD-related problems, such as carotenoids. These are, in fact, natural pigments with notable antioxidant and anti-inflammatory properties. For example, β-carotene has demonstrated anti-inflammatory activity in animal models of DSS-induced ulcerative colitis [198]. Among the carotenoid xanthophylls, the most interesting for antioxidant activity is AXT, produced by various algae and microorganisms [199]. L’AXT (3,3′-dihydroxy-β,β-carotene-4,4′-dione) is a highly effective keto-carotenoid belonging to the class of xanthophylls. Its chemical structure comprises two hydroxyl groups and two carbonyl groups, which give it a powerful antioxidant activity: it is in fact 10 to 550 times more effective than other antioxidants such as vitamin E, β-carotene and lycopene in neutralizing free radicals [199,200,201]. Its chemical nature makes it very stable and lipophilic, thus increasing its bioavailability and accumulation in biological systems [202,203]. This carotenoid is biologically synthesized by microalgae, in particular *Haematococcus lacustris*, which can accumulate AXT up to 4–5% of the dry weight under stress conditions (high light intensity or nutrient scarcity) [204,205,206]. In addition to these green algae, AXT is also found in some yeasts (e.g., *Phaffia rhodozyma*), some mushrooms (*Xanthophyllomyces dendrorhous*), and several seafoods such as salmon and shrimp [207,208,209,210,211]. Other microalgae, such as *Chlorella zofingiensis* and *Scenedesmus* sp., have also been studied, but produce lower amounts of AXT [131]. Due to its bioactivity, AXT is of great interest in the pharmaceutical and nutraceutical sectors, with applications ranging from the treatment of inflammation to use as a food supplement [100,212,213]. Recent technological advances in cultivation processes have allowed the optimization of biomass production and AXT yield [214]. Furthermore, genetic modification processes have been employed to optimize AXT biosynthesis pathways in microalgae, with the aim of increasing yield and reducing production costs [215]. AXT is a highly effective antioxidant, able to neutralize free radicals and reduce oxidative stress, thus effectively preventing cellular damage in long-term chronic diseases. Furthermore, it possesses anti-inflammatory effects, as demonstrated by numerous in vitro and in vivo studies, acting on a broad spectrum of chronic diseases [212,214,216,217,218]. Its action is mainly expressed through the interaction with key cellular pathways such as NF-κB, MAPK, JAK-STAT and Nrf2, which regulate both the inflammatory response and antioxidant defense mechanisms [219,220]. In some studies, the role of AXT as a protective agent in the treatment of diseases, including colitis, has been investigated: in murine models, astaxanthin modulated inflammatory responses and preserved the intestinal barrier function [217,218]. Positive effects of AXT have also been observed at the cardiovascular level, with improvements in lipid metabolism, endothelial function and a reduction in the risk of atherosclerosis, as confirmed by a meta-analysis [205,221]. Its ability to inhibit the expression of the metalloproteinase MMP-9 and to reduce lipid deposits in macrophages make it an ideal candidate for the prevention of atherosclerosis [222,223,224]. The neuroprotective role of AXT is of particular interest, since it is able to preserve cognitive functions and improve synaptic plasticity, as demonstrated by studies conducted on young and old mice [225]. The modulation of neuroinflammatory pathways and the reduction of oxidative stress suggest a possible therapeutic use of AXT in neurodegenerative diseases [226,227]. In models of Alzheimer’s disease and Parkinson’s disease, astaxanthin has shown protective effects, reducing neuroinflammation and neuronal apoptosis, thanks to the activation of pathways such as PI3K/Akt and Nrf2 [222,226,228,229,230,231,232,233,234]. By selectively inhibiting NF-κB activation by reducing IκB-α degradation, thus preventing subsequent nuclear translocation of NF-κB, the transcription of key pro-inflammatory genes such as TNF-α, IL-1β, IL-6, COX-2 and iNOS is blocked [231,232,234,235,236,237]. Studies conducted on cellular and animal models indicate that AXT is particularly effective in inhibiting the chemokine MCP-1, responsible for macrophage recruitment, as well as modulating the immune response by affecting the balance between Th1 and Th2 [219,238,239]. In parallel, AXT acts as a potent antioxidant, both directly, by neutralizing free radicals, and indirectly, through the activation of the Nrf2 signaling pathway and the expression of antioxidant enzymes such as superoxide dismutase (SOD) and glutathione peroxidase, thus protecting cells from oxidative stress [151,165,166,167]. Experimental studies have confirmed these properties and, in particular, treatments with AXT have shown a clinically significant reduction in the levels of C-reactive protein (CRP) and 8-OHdG (8-hydroxy-2′-deoxyguanosine), a marker of oxidative DNA damage [240,241]. Furthermore, positive regulations of the immune response, such as the activity of T and B lymphocytes, have also been observed [199,242]. AXT represents a promising therapeutic candidate for the treatment of IBD [238]. Its molecular structure has been shown to be important in neutralizing reactive oxygen species (ROS) and reducing oxidative stress, decreasing inflammation and redox imbalance as well as related intestinal damage [208,209,243,244]. In vitro studies have shown that AXT reduces oxidative damage (measured by lipid peroxidation levels) and, at the same time, increases the activity of antioxidant enzymes such as SOD and catalase, thus protecting intestinal epithelial cells from oxidative damage [243,245,246]. The mechanisms of action of AXT, based on the inhibition of NF-κB, a key factor in the release of pro-inflammatory cytokines such as TNF-α and other interleukins, show a decrease in key inflammatory biomarkers, which reflects and confirms an additional anti-inflammatory action [208,209,243,247]. Furthermore, AXT promotes the activation of the transcription factor Nrf2, which protects against oxidative damage and stimulates the expression of detoxifying and antioxidant enzymes, thus providing an additional level of protection during inflammatory processes [243]. As mentioned above, the action of AXT has also been associated with the modulation of important interleukin pathways, such as TNF-α, IL-1, IL-6, IL-17 and IL-23 [209,248]. Kim et al. have shown that AXT treatment reduced the production of TNF-α and IL-6 in macrophages, thanks to the inhibition of the NF-κB pathway [249]. AXT has also been observed to reduce the overproduction of IL-1β, a pro-inflammatory cytokine crucial in the pathogenesis of IBD [250]. AXT action has shown a significant effect on the differentiation of helper T cells (Th) in IBD, modulating the ratio between Th1 and Th2 responses [251,252]. In IBD, in fact, a dominant Th1 response is generally associated with colonic inflammation, while a deviation towards Th2 favors tissue repair and damage interruption [72,253,254]. Other observations have highlighted an immunomodulatory activity, suppressing pro-inflammatory cytokines such as TNF-α and inducing the production of anti-inflammatory cytokines such as IL-4 and IL-10 [123,219]. These data suggest that AXT is an effective therapeutic agent for the treatment of IBD, thanks to its ability to selectively modulate T cell differentiation and cytokine profile [123,219]. Its biological activity is expressed not only through a protective antioxidant mechanism but also through a balancing system between Th1 and Th2 responses, relevant for the reduction of IBD-associated inflammation [123,218,219]. AXT also acts on intestinal epithelial cells, helping to preserve the integrity of the intestinal barrier [255,256]. Indeed, the benefits of AXT include improving intestinal barrier function and strengthening epithelial cells to maintain the integrity of tight junctions, thus preventing increased intestinal permeability [123,257,258]. Its influence on the expression of proteins such as occludin and claudin strengthens barrier function, preventing the onset of IBD symptoms and reducing the severity of the disease, through the inhibition of mucosal erosion and ulceration [245,246]. The action of AXT allows the maintenance of the mucosal barrier, essential to prevent the translocation of antigens and pro-inflammatory agents from the intestinal lumen to the underlying tissues [255,259,260]. Numerous in vitro and in vivo models have confirmed its anti-inflammatory and protective effects against colitis [123,261,262]. In animal models, supplementation with AXT inhibited the development of colitis symptoms, significantly reducing the expression of pro-inflammatory cytokines such as TNF-α and IL-6, improving colonic histology and increasing mucosal integrity [123,205,261,262,263]. AXT treatment also reduced disease activity and mucosal damage in mice with DSS-induced colitis, correlated with lower levels of oxidative stress markers and pro-inflammatory cytokines [123,205,224,261,262,263,264]. Finally, AXT reduces NO release from macrophages, as excessive NO production can prolong inflammation and increase tissue damage, reducing oxidative stress and the resulting inflammatory burden [235,249]. Further confirmation shows that AXT treatment led to a marked decrease in inflammatory biomarkers and the activation of proteins involved in the formation of tight junctions in colonic epithelial cells, further strengthening its protective effect against inflammation and oxidative stress [265,266,267]. The positive effects of AXT use in IBD have also focused on its effects on the microbiota. Fang Liu et al. showed that krill oil, rich in ω-3 PUFA and AXT, modulates the intestinal microbiome and metabolome by attenuating inflammation and promoting the resolution of colitis in in vitro and in vivo models and normal intestinal eubiosis [268]. In several in vivo studies on murine models, AXT obtained from algal extracts promoted the propagation of beneficial microbial populations such as *Bacteroidia*, *Bacilli*, *Clostridia*, and *Verrucomicrobia* in the intestine, modulating microbial signatures and regulating metabolic homeostasis [269,270]. Wang et al. investigated not only the therapeutic but also preventive effects of AXT which, with the additional use of probiotics, prevented intestinal dysbiosis, oxidative stress, inflammation, and metabolic disorders, all key factors in IBD [270,271]. In murine models of diet-induced obesity, AXT improved lipid profiles and liver function, increased the activity of antioxidant enzymes, and reduced levels of reactive oxygen species. In parallel, a modulation of the intestinal microbiota has been observed, with an increase in microorganisms with beneficial effects on intestinal homeostasis [271]. Other evidence has highlighted the role of AXT as a modulator of numerous intracellular pathways related to oxidative stress and inflammation, such as NF-κB, Nrf2, PI3K/Akt, JAK-STAT, and MAPK signaling, reducing the production of pro-inflammatory cytokines, increasing the antioxidant response, and limiting apoptotic processes [231,272]. Finally, AXT has been shown to be useful in preserving the integrity of the intestinal mucosa in immunosuppressed mouse models, reducing oxidative stress and promoting a more balanced microbial flora, with higher levels of beneficial bacteria and an increased production of short-chain fatty acids [272]. AXT, therefore, has demonstrated promising therapeutic potential for regulating IBD symptoms and related pathologies, with a pronounced protective activity of the intestinal environment [123,273,274]. In conclusion, AXT is an interesting candidate for the treatment of IBD, thanks to the regulation of interleukin pathways, the strengthening of the intestinal barrier, and the suppression of NO production in macrophages [244,264,275]. Evidence suggests that AXT could be used as a dietary adjuvant alongside current IBD therapies [276]. The numerous mechanisms of action of AXT—in particular its antioxidant and anti-inflammatory activity—confirm its potential as a therapeutic agent for the management of IBD [238,244]. In recent years, the therapeutic role of microalgae in modulating the intestinal microbiota as a source of prebiotics has been emerging, thanks to their rich nutritional profile. Bioactive compounds derived from microalgae include polysaccharides, bioactive proteins and peptides, sterols, terpenoids, polyphenols and cyclic polysulphide compounds. Many studies highlight those microalgae activate the immune system, protect the defensive capacity and increase anti-infective activities. Microalgae derivatives have antiviral, antibacterial, anticancer, immunomodulatory and antioxidant properties [277]. Among the most important microalgal derivatives with high antioxidant properties are terpenes [278]. Studies show that β-carotene is capable of suppressing lipid peroxidation, inflammation caused by oxidative stress, and tumour formation [279]. For example, Red algae produce halogen, a monoterpene with high anti-tumour activity [280]. In addition, microalgae produce high amounts of vitamins, including α-tocopherols, which, in addition to being the most potent antioxidants, play a key role in the prevention of neurodegenerative diseases [279]. Short-chain fatty acids (SCFAs) play a very important role in the interaction between the microbiome and the immune system. These metabolites are produced during the colon filtration process by the microbiota. In an in vitro simulated gut model, the fermentation activity of gut microbes of the microalgae *Chlorella vulgaris* and its compounds increased SCFA production. Studies show that the presence of SCFAs, particularly acetic, propionic, butyric and isobutyric acids, produced by beneficial gut bacteria, is indicative of good health. A decrease in bacteria causing dysbiosis, such as Staphylococcus, Enterococcus and Enterobacteriaceae, has been observed with fatty acid production [281]. On the other hand, polyunsaturated fatty acids (PUFAs) produced by microalgae, especially omega-3 and DHA, have attracted considerable interest in the potential treatment of IBD. PUFAs have been observed to block inflammation, normalise the immune response, and protect intestinal epithelial cells, thereby helping to maintain microbiome balance [273]. Other microalgal compounds with therapeutic potential for IBD include tocopherols and phycobiliproteins. Furthermore, the therapeutic effect of phycocyanin and phycocyanobilin has been studied in mouse models of colitis, demonstrating anti-inflammatory and antioxidant activity. The inhibitory effect of phycocyanin and phycocyanobilin on IgE activity, reduction of vascular permeability and inflammation through low expression levels of inflammatory genes and pro-inflammatory cytokines (TNF-α) in the gut and systemic circulation was observed [281]. IBD represents an interesting model for the complex interactions between the microbiota and the immune system in the context of chronic inflammation. In this regard, microalgae, thanks to their high levels of bioactive compounds, have emerged as agents for managing inflammatory conditions. Some microalgae species, such as *Spirulina platensis*, *Chlorella vulgaris*, and *Phaeodactylum tricornutum*, are rich in bioactive compounds such as phycocyanin, polysaccharides, polyphenols, and unsaturated fatty acids, with anti-inflammatory, antioxidant, and immunomodulatory properties and useful for managing IBD [281,282]. Among the most commonly present compounds, AXT is a promising candidate for modulating inflammation and oxidative stress. *Haematococcus lacustris* is currently the main natural source of this carotenoid, representing a significant percentage of its dry weight.

The possibility of engineering microalgae to produce larger quantities of metabolites makes these organisms of particular scientific and biotechnological importance. Clinical studies have shown that oral administration of astaxanthin is the most common, with a recommended dose of 4–12 mg to evaluate its anxiolytic effects, which can be adjusted depending on the desired effect [133]. Clinical studies have also shown that doses of 12–24 mg have been used without signs of marked toxicity.

Preliminary studies suggest that dietary supplementation with microalgae may promote interactions with the microbiota and stimulate the development of anti-inflammatory molecules derived from fermentation processes. Recent studies have suggested that, despite the high carbohydrate content of seaweed and microalgae, most of them are not digested in the gastrointestinal tract and therefore act as dietary fiber. Alginates, fucoids, carrageenans, xylans, etc., are soluble fibers that are not completely fermented by the microbiota into short-chain fatty acids, despite their ability to positively regulate intestinal metabolism. Other algal polysaccharides (EPs) can instead act as insoluble fibers (cellulose, mannans, β-glucans, lignin, etc.). Microalgal PSs may provide health benefits and can be used as emerging prebiotics [283].

In recent years, oxylipins have attracted the attention of the scientific community. These molecules are derived from the enzymatic or non-enzymatic oxidation of PUFAs and play a dual role in IBD. While some species are pro-inflammatory, others, such as the resolvins discovered by Serhan et al., actively promote its resolution [181]. Pro-resolving oxylipins, in fact, modulate the immune system and the intestinal microbiota, as evidenced by an in vivo study by Arita et al. [284]. In this context, microalgae represent a sustainable source of precursor fatty acids and a natural reservoir for novel lipid metabolites such as oxylipins, offering a nutritional strategy to modulate inflammation and the microbiota [285,286].

## 9. Conclusions

The gut microbiota plays a key role in the proper functioning of the body. Several studies have confirmed how the microbiota influences the onset, rate of progression and treatment of tumors. The interaction of the microbiota and cells of the immune system generates a microenvironment that when altered causes loss of homeostasis and tumor initiation. Tumors develop through cells that ignore tolerance rules and escape tumor surveillance mechanisms. Studies have identified defensive mechanisms by the microbiota such as antigen mimicry and transformation of chemotherapeutic agents. Despite numerous studies, the microbiota–immune system–tumor interaction presents complex mechanisms that bring different research teams to attention. Insight into these processes would lead to a clearer understanding and better management of treatments.

## Data Availability

No new data were created or analyzed in this study.
